# Real World Use of an Internet Intervention for Pediatric Encopresis

**DOI:** 10.2196/jmir.1081

**Published:** 2008-06-30

**Authors:** Lee M Ritterband, Kaveh Ardalan, Frances P Thorndike, Joshua C Magee, Drew K Saylor, Daniel J Cox, James L Sutphen, Stephen M Borowitz

**Affiliations:** ^3^Department of PediatricsUniversity of Virginia Health SystemCharlottesvilleVAUSA; ^2^School of MedicineUniversity of Virginia Health SystemCharlottesvilleVAUSA; ^1^Department of Psychiatry and Neurobehavioral SciencesUniversity of Virginia Health SystemCharlottesvilleVAUSA

**Keywords:** Internet, behavioral intervention, encopresis

## Abstract

**Background:**

The Internet is a significant source of medical information and is now being shown to be an important conduit for delivering various health-related interventions.

**Objective:**

This paper aimed to examine the utility and impact of an Internet intervention for childhood encopresis as part of standard medical care in a “real world” setting.

**Methods:**

Patients diagnosed with encopresis were given a Web-based information prescription to use an Internet intervention for pediatric encopresis. A total of 22 families utilized the intervention between July 2004 and June 2006. A chart review and phone interview were undertaken to collect user characteristics; defecation-related information, including frequency of soiling, bowel movements (BMs) in the toilet, and amount of pain associated with defecation; and information on computer/Internet usage. Three questionnaires were used to examine the utility of, impact of, and adherence to the Internet intervention. Program utilization was obtained from a data tracking system that monitored usage in real time.

**Results:**

Overall, parents rated the Internet intervention as enjoyable, understandable, and easy to use. They indicated that the Internet intervention positively affected their children, decreasing overall accidents and increasing child comfort on the toilet at home. Of the 20 children who initially reported fecal accidents, 19 (95%) experienced at least a 50% improvement, with a reduction of accident frequency from one fecal accident per day to one accident per week. Although it is not clear whether this improvement is directly related to the use of the Internet intervention, patient feedback suggests that the program was an important element, further establishing Internet interventions as a viable and desirable addition to standard medical care for pediatric encopresis.

**Conclusions:**

To our knowledge, this is the first time a pediatric Internet intervention has been examined as part of a “real world” setting. This is an important step toward establishing Internet interventions as an adjunctive component to treatment of pediatric patients in a clinical setting, particularly given the positive user feedback, possible cost savings, and significant potential for large-scale dissemination.

## Introduction

The Internet has become a vital source of health care and medical information. Approximately 113 million Americans have searched for health-related information on the Internet [1], and a majority of children and adolescents are now online [2]. Parents are more likely to use the Internet than are nonparents, with 83% of adults with a child using the Internet compared to 60% of adults without a child at home [3]. While the vast majority of health-related websites are informational [4,5] a growing number of sites provide health interventions that patients can use to self-treat or use in conjunction with face-to-face treatment [6]. Such Internet interventions are typically behaviorally based treatments that have been operationalized and transformed for delivery via the Internet. They are usually based on empirically validated, face-to-face interventions and are enhanced by graphics, animations, audio, and video. These interventions are generally interactive, highly structured, self-guided or semi-self-guided, personalized to the user, and tailored to provide follow-up and feedback [7].

There is a growing literature on the feasibility and efficacy of Internet interventions for a variety of pediatric disorders, including body image/disordered eating [8-11], weight loss, nutrition, physical activity [12-17], encopresis [18], asthma [19,20], smoking [21,22], pain [23], and traumatic brain injury [24-26]. While studies have shown that Internet interventions can be used to successfully treat a diverse set of pediatric disorders, “real world” use of these interventions (defined as patients being given access to these programs as part of their clinical care as opposed to part of a research study) has not been examined.

In the real world, Internet interventions will likely be sought out directly by consumers or prescribed by a clinician. Clinicians can direct patients and families to Internet interventions using a Web-based information prescription. An information prescription is a “prescription of focused, evidence-based information...to manage a health problem” [27]. We have previously shown that 65% of individuals (77% who receive an email reminder) will visit a website specifically prescribed by their clinician [28]. There are no data, however, that show how patients use and react to the prescription of an Internet intervention within a “real world” setting.

Between 1.5% and 7.5% of children suffer from encopresis [29]; 25% of visits to pediatric gastroenterology clinics [30] and 3% of visits to general pediatric clinics are due to encopresis [31]. In this paper, we examine the utility and impact of an Internet intervention for pediatric encopresis prescribed as part of standard medical care for patients seeking treatment for encopresis at a pediatric gastroenterology clinic in a major medical center. To our knowledge, this is the first attempt to examine the prescription of a pediatric-based Internet intervention as part of standard medical care. It is important to note that this was not a randomized controlled trial, but rather an attempt to examine the use of an Internet intervention as part of clinical care (not as part of a research study) by retrospectively reviewing medical records and conducting phone interviews. While outcome data on defecation related variables were collected and are reported here, improvements in this area cannot be directly attributed to the Internet intervention. Rather, this study examines the users’ perceived impact of the prescribed intervention in the context of their standard medical care.

## Methods

### Patients

Patients included families with an encopretic child seen at the Pediatric Gastroenterology Clinic at the University of Virginia Children’s Hospital. All children had a documented diagnosis of encopresis, as noted in their medical record, and had been given access to the pediatric encopresis Internet intervention as part of treatment.

### Procedure

As part of treatment, the pediatric gastroenterologist seeing the children (SB or JS) provided families with a Web-based information prescription directing them to U-CAN-POOP-TOO, an Internet-based intervention for childhood encopresis (described below). The family provided the gastroenterologist with their email address, and an email message was sent to them with instructions on how to begin using the program.

Patients were seen between July 2004 and June 2006 and were contacted for an interview between June and August 2006 (conducted by KA). This interview occurred anywhere from 2 months to 2 years following their appointment. Relevant patient data were available from the Internet intervention data tracking system (usage data) and medical charts. Consent was obtained at the beginning of the phone interview. This protocol was approved by the University of Virginia Health System Institutional Review Board.

### Measures

Data came from three sources: (1) medical charts, (2) the U-CAN-POOP-TOO data tracking system, and (3) a phone interview. The medical chart provided basic demographic and descriptive information, including patient characteristics, contact information, and diagnoses. It also provided history and frequency of soiling, frequency of bowel movements (BMs) in the toilet, and amount of perianal pain the child experienced during defecation. The U-CAN-POOP-TOO data tracking system contained usage statistics of the Internet intervention for each patient, including the number of completed program components.

During the phone interview, the parents were asked questions about the following: additional user characteristics (eg, school grade, developmental delays), retrospective and current bowel-related information (frequency of accidents, BMs on toilet, and pain ratings), and computer/Internet use (how often an individual uses a computer and the Internet as well as their comfort level with both). Three structured questionnaires were completed during the interview. The phone interview also included open-ended questions about what parents believed were the most helpful and least helpful components of the program. The three questionnaires, developed mostly for this interview, included the following:

1. U-CAN-POOP-TOO Utility Questionnaire: This inquired about the extent to which the parent and child found the program useful, enjoyable, understandable, and easy to use. There are 10 items, 8 requiring responses on a 5-point scale from 1 (“not at all”) to 5 (“very”), and 2 items asking what the most and least helpful aspects of the Internet program were. For the 8 Likert scale items, the alpha coefficient was .69, indicating good internal reliability. It was administered to all parents who had used the U-CAN-POOP-TOO program.
2. U-CAN-POOP-TOO Impact Questionnaire: This asked parents to rate how much they perceived the program helped their child. There are 25 items, and responses are on a 5- point scale from 1 (“not at all”) to 5 (“very”). Parents could also respond with a 0 to indicate that the item was not relevant to them. To establish internal reliability, the items were broken down into five categories, including physical symptoms (alpha = .88), comfort (alpha = .80), worry/mood (alpha = .65), school/social support (alpha = .94), and cost/time (alpha = .64).The questionnaire was administered to all parents who had used the U-CAN-POOP-TOO program.
3. Internet Intervention Adherence Measure: This measure attempts to identify obstacles that interfered with the patient completing the program. Obstacles are categorized as Internet/computer/technical issues, personal/family issues, intervention-general issues, and intervention-specific issues. Patients are asked to respond to the 35 items on a 3-point scale from 1 to 3, indicating whether that obstacle had “no part,” “a little part,” or “a major part” in why they stopped using the program. The measure was administered to patients who stated that they stopped using the U-CAN-POOP-TOO program for some reason other than that their problem was “resolved.” This is an expanded and more detailed measure to the one we used in a previously published paper examining barriers to following through with a Web-based information prescription [28].

### Internet Intervention for Pediatric Encopresis (U-CAN-POOP-TOO)

**Figure 1:**
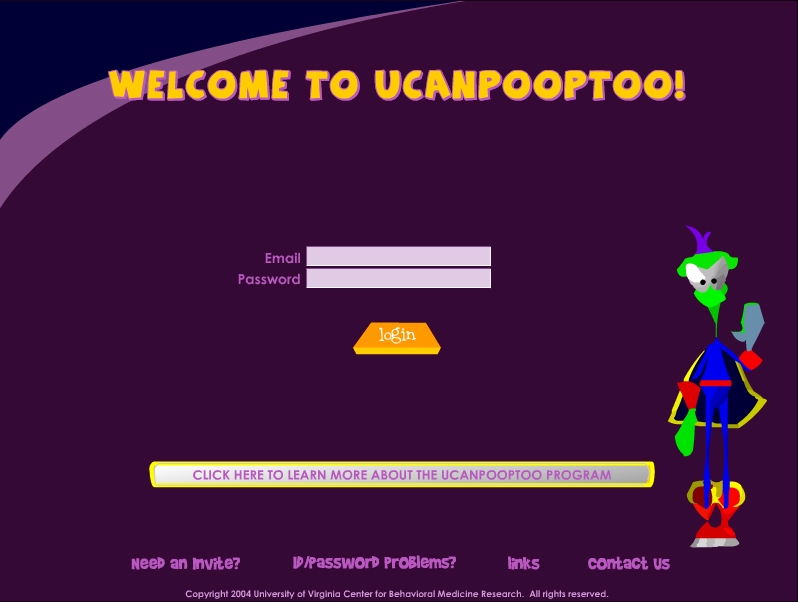
Screenshot of the “Welcome” page of U-CAN-POOP-TOO

**Figure 2:**
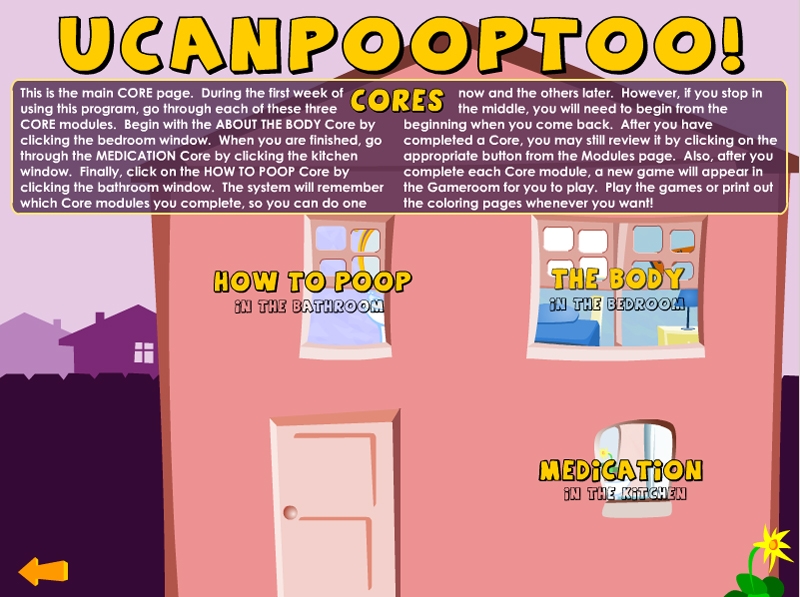
Screenshot of the “Welcome” page of U-CAN-POOP-TOO

The U-CAN-POOP-TOO program (Figure 1) was developed for the treatment of pediatric encopresis and has been found in a randomized controlled trial to be an effective addition to standard medical care [18]. The child-focused program targets primarily 5- to 12-year-olds, but it was designed to be used by the child and parent(s) together. Using graphics and animation, detailed information is presented through intensive and engaging tutorials. Users are educated about anatomy, physiology, and pathophysiology of digestion (Anatomy Core); clean-out and laxative treatments (Medication Core); and behavioral techniques for treatment of encopresis (Behavior Core). The three core modules of the program (Figure 2) take 60 to 90 minutes to complete, and all users are instructed to review them during the first week. New modules are assigned each week based on a follow-up assessment the user completes about their child’s status. Not all modules are necessarily viewed by all users; only those modules identified as relevant are assigned and encouraged to be reviewed. However, all modules can be viewed by all users. The follow-up is comprised of 17 to 20 questions, depending on the week. The system contains a total of 22 modules, each which takes 5 to 10 minutes to review. See Ritterband et al (2003) for a more detailed description of the program [18].

### Statistical Methods

Descriptive statistics of the 22 subjects included in the data analysis were first computed, including gender, race, age, and education as well as developmental delays, accident history, and the age of the child when toilet training was completed. Repeated-measures analysis of variance and correlations were calculated to examine changes in the main bowel-related variables of interest. Cure and success rates were also computed. Additional descriptive statistics were computed to explore program usage patterns by patients. To examine the impact of computer/Internet usage specifically, a composite *z* score was created for each patient by combining the patient’s email and Internet usage. This composite *z* score was generated by computing a *z* score for each patient by comparing him or her to the overall group mean on each variable (number of standard deviations from the overall mean). The email and Internet *z* scores for each person were then averaged to calculate the composite score. Pearson correlations were computed between the computer/Internet usage *z* scores and the initial to follow-up change scores. Descriptive statistics were calculated based on parents’ responses to the measures of perceived utility and helpfulness of the program, as well as perceived obstacles to completing the program. Finally, responses to the open-ended questions about the least and most helpful aspects of the program were reviewed for clear themes.

## Results

### Patient Characteristics

**Figure 3:**
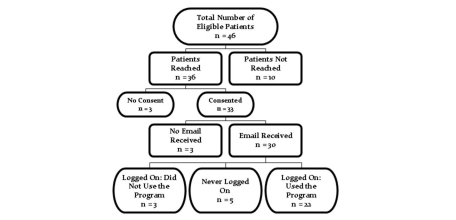
Flowchart of patient enrollment

Between July 2004 and June 2006, 46 patients seen in the pediatric gastroenterology clinic for encopresis were provided the U-CAN-POOP-TOO Web-based information prescription. Figure 3  shows the flow of patients: 10 patients could not be reached by phone or email for the interview; of the remaining 36 patients, 3 did not provide consent, 3 stated that they never received the initial email with their personalized log-in information, 5 never logged on, and 3 logged on but never viewed any of the intervention material. No subsequent data were collected on these patients. This resulted in 22 patients (13 males and 9 females). See Table 1 for a summary of patient characteristics.

**Table 1:** Patient characteristics (N = 22)

Characteristic	
Gender	
Male	13 (59%)
Female	9 (41%)
Race	100% Caucasian
Age	
Range	5 years, 1 month to 12 years, 11 months
Mean (SD)	8 years, 10 months (2 years, 3 months)
Education	
Range	kindergarten to 5th grade
Median	3rd grade
Developmental delays^*^	3 (14%)
Accident history (duration of encopresis), mean (SD)	44.73 months (26.27 months)
Age of toilet training, mean (SD)^†^	33.62 months (12.86 months)

^*^These were based on self-report and were identified as “mild neuromotor processing abnormality,” “fine motor skill problems (in occupational therapy),” and “cerebral palsy.”

^†^Indicates missing data from initial chart review (N = 21).

### Bowel-Related Statistics

Three main bowel-related variables were examined for the initial period and the follow-up period: (1) the number of fecal accidents over a 2-week period, (2) the number of BMs passed in the toilet over a 2-week period, and (3) the average amount of perianal pain experienced during defecation over a 2-week period, based on a 3-point Likert scale from 0 (“none”) to 2 (“a lot”). The initial period was the 2 weeks before the children were enrolled in the program, and the follow-up period was the 2 weeks immediately before the phone interview.

The number of accidents decreased from 13.86 (SD 10.40, median 13.00) during the initial period to 2.14 (SD 2.21, median 1.00) during the follow-up period (F_1,21_ = 27.29, *P* < .001). No significant changes were found for the number of BMs in the toilet (F_1,20_ = .01, *P* < .93) or the amount of pain the child experienced during defecation (F_1,17_ = 2.84, *P* < .12). These results are summarized in Table 2.

**Table 2:** Initial to follow-up bowel-related statistics (N = 22)

	Initial (Chart Review) Mean (SD)	Follow-Up (Interview) Mean (SD)	*P*
Accident frequency (per 14 days)	13.86 (10.40)	2.14 (2.21)	.001
BMs in toilet (per 14 days)	14.62 (10.68) (N = 21)^*^	14.82 (8.65)	.93
Pain on defecation	.56 (.78) (N = 18)^*^	.14 (.47)	.12

^*^Indicates missing data from initial chart review.

While all patients included in the analyses had a diagnosis of encopresis, two of the 22 patients reported no accidents in the 2 weeks prior to using the system. These same two patients continued to be accident free during the follow-up period. Of the remaining 20 patients, 10 (50%) reported having no more than one accident in the 2 weeks prior to the phone interview. Four patients (20%) were considered “cured” by indicating that they had no accidents during the follow-up period. All but one of the 20 patients (95%) had at least a 50% reduction in accident frequency from the initial to interview period. The median reduction was 7.5 accidents in 2 weeks, supporting the notion that these were substantive improvements.

The number of fecal accidents in the 2-week initial period did not predict the number of accidents at the follow-up period. That is, there were no significant correlations between initial and follow-up periods for accident frequency (*r* = .05, *P* < .84, N = 22), BMs passed in the toilet (*r* = .27, *P* < .24, N = 21), or amount of pain with defecation (*r* = −.10, *P* < .71, N = 18), suggesting that the severity of the symptoms at the time of enrollment did not relate to how much the patient benefited from treatment.

### U-CAN-POOP-TOO Use Statistics

 Of the 22 patients who used U-CAN-POOP-TOO, 18 (82%) completed all three assigned cores (main treatment components). All 22 patients completed the Anatomy Core; 20 completed the Medication Core; and 18 completed the Behavior Core. A total of 12 patients (55%) completed one follow-up, four (18%) completed a second and third follow-up, and two of these four (9%) completed more than three follow-ups. Modules were individually assigned based on responses to follow-ups; however, patients had access to all the modules. The average number of modules completed was 7.23 (SD 9.64); 14 patients (64%) completed at least one module.

There was significant variability in the amount of time elapsed between when patients were initially given access to U-CAN-POOP-TOO (between July 2004 and June 2006) and the time the phone interview was conducted (between July and August 2006). To examine whether time alone may have been a significant factor in terms of reported encopretic symptoms, patients were divided into three time-based groups with an attempt to make cell sizes roughly even: (1) those enrolled between July 2004 and June 2005 (N = 5), (2) those enrolled between July 2005 and December 2005 (N = 7), and (3) those enrolled between January 2006 and June 2006 (N = 10). No differences were found among these three time groups for changes in accident frequency (F_2,19_ = 1.93, *P* < .18), BMs in the toilet (F_2,18_ = 1.54, *P* < .25), or pain experienced during defecation (F_2,15_ = 1.57, *P* < .25).

### Computer/Internet Use

The 22 families reported checking their email 13.18 times per week (SD 14.03) and using the Internet 10.39 hours per week (SD 10.15). On average, they indicated their comfort level using the Internet to be 2.64 (SD .73) on a 5-point scale ranging from 0 (“not at all comfortable”) to 4 (“I’m an expert”). A total of 15 of the 22 families (68%) had high-speed Internet access at home, six (27%) had dial-up access, and one was unsure about the connection speed. The above variables (computer/Internet usage, Internet comfort, and connection speed) were examined to determine whether they affected outcome. No significant correlations were found between computer/Internet usage and the change from initial to follow-up period for accident frequency (*r* = .09, *P* < .69, N = 22), BMs passed in the toilet (*r* = .38, *P* < .09, N = 21), or amount of pain associated with defecation (*r* = .08, *P* < .76, N = 18). Internet comfort and connection speed were also not significantly correlated to changes in any of the bowel-related outcome variables (*r* values ranged from −.17 to .27; *P* values ranged from .25 to .59).

### Utility of U-CAN-POOP-TOO

In general, parents reported favorable reactions to U-CAN-POOP-TOO. They tended to like the program (mean 4.62, SD 0.50, N = 21) and found it understandable (mean 5.00, SD 0.00, N = 20) and easy to use (mean 4.62, SD 0.74, N = 21). They also believed that their child liked the program (mean 4.05, SD 1.28, N = 21) and found it understandable (mean 4.32, SD 0.89, N = 19) and easy to use (mean 4.47, SD 0.77, N = 19). Those who responded “not applicable” to items on the U-CAN-POOP-TOO Utility Questionnaire were not included in the analysis for that item (explaining the varying sample sizes). In addition to questions about enjoyment, comprehension, and ease of use, parents were also asked what they believed were the most helpful and least helpful components of the program. They found the tutorials about anatomy and pathophysiology to be one of the most helpful aspects of the program. They also liked that the program was geared toward the child, but that it was comprehensive and nonjudgmental. No clear themes emerged from the “least helpful” question.

### Impact of U-CAN-POOP-TOO

The U-CAN-POOP-TOO Impact Questionnaire was administered to examine how much the parents believed the program affected outcome. Those who responded “not applicable” were not included in the analysis for that item. On average, 19 out of 25 items (76%) were rated at least “somewhat helpful,” and no item was described as “not at all helpful.” On the 1- to 5-point scale, average responses ranged from a low of 2.33 (the program helped reduce the number of times parents had to remind their child to use the bathroom) to a high of 4.2 (the program helped the child feel more comfortable using the toilet at home). See Table 3 for a listing of individual items.

**Table 3:** U-CAN-POOP-TOO Impact Questionnaire

Question: How much did the U-CAN-POOP-TOO program help (from 1 “not at all” to 5 “very”)	No.^*^	Mean (SD)
**Physical Symptoms**		
Decrease the number of overall accidents	17	3.71 (1.21)
Decrease the number of accidents at school	14	3.43 (1.28)
Decrease the number of accidents at home	16	3.56 (1.26)
Increase the number of times your child goes to the bathroom on his/her own	17	2.94 (1.39)
Reduce the number of times you, the parent, had to remind them to use the bathroom	18	2.33 (1.46)
Decrease the use of diapers during the day	7	3.00 (1.29)
Decrease the use of diapers during the night	5	2.80 (1.20)
Increase number of BMs in the toilet	17	3.65 (1.27)
Your child have less pain with defecation	9	3.44 (1.13)
Improve your child’s appetite	9	3.33 (1.41)
Reduce your child’s stomach pain	12	3.17 (1.19)
**Comfort**		
Your child feel more comfortable using the toilet at home	20	4.20 (1.01)
Your child feel more comfortable using the toilet at school	15	2.67 (1.59)
Your child feel more comfortable using the toilet out (restaurants, mall, etc)	16	2.69 (1.66)
**Worry/Mood**		
Reduce your child’s worry about something ‘bad’ happening when s/he is on the toilet	10	3.20 (1.40)
Reduce your child’s worry about having a BM; ie, worried about pain or stool not coming out	10	3.50 (1.08)
Reduce your child’s worry about having accidents	16	3.50 (1.16)
Improve your child’s mood (happier, more confident)	17	3.41 (1.18)
**School/Social**		
Increase school attendance	3	3.00 (1.73)
Improve school performance	7	3.00 (1.41)
Improve participation in sports and social activities, like scouts, visiting friends, religious groups	13	2.46 (1.20)
Improve peer relationships/friendships	14	3.00 (1.18)
Improve relationships with family	17	3.59 (1.37)
**Related Cost/Time**		
To what extent do you believe this Internet intervention helped reduce the number		
…of visits with your doctor/doctor’s office?	14	3.07 (1.27)
…of phone calls with your doctor/doctor’s office?	14	3.14 (1.70)

^*^Those who responded “not applicable” were not included in the analysis for that item (explaining the varying sample sizes).

### Adherence

Of the 22 patients examined, 16 indicated that they stopped using the program for some reason other than that their problem was “resolved.” They were administered the Internet Intervention Adherence Measure, the questionnaire used to identify obstacles to using the program. Based on the responses, only two items had a mean score of 2 or greater (on a 1- to 3-point scale). They were “I just forgot [to go to the website]” (mean 2.00, SD 0.89) and “I didn’t have time in my schedule” (mean 2.06, SD 0.85). Notably, these were the same top two obstacles identified in our previous study examining the use of Web-based information prescriptions [27].

## Discussion

This paper examined the utility and impact of an Internet intervention for childhood encopresis provided as a Web-based information prescription in a “real world” situation. Based on parent participant report, there was an almost universal belief that the system had a substantive and positive effect on their child. When parents were asked to rate their perception of the impact of the Internet intervention, they indicated that the Internet program helped decrease the number of accidents and increase the child’s comfort in using the toilet at home. Parents also believed that the system helped reduce their child’s physical symptoms of encopresis and level of worry, improved their child’s mood, and increased and improved their child’s school and social activities. Additionally, parents believed that U-CAN-POOP-TOO helped reduce the number of calls and visits to their doctor, implying that there may be cost reductions with the use of the program.

Accompanying improvements in defecation-related variables were reported, including a marked decrease in fecal accident frequency from the initial to follow-up period in this sample of patients. However, it cannot be determined if this improvement is directly attributable to the Internet intervention due to the major limitation of not having a control group. This precludes reaching a definitive conclusion as to whether the Internet intervention caused the improvement. Yet, while the lack of a control group makes it impossible to state that the intervention led to the observed improvements, parents clearly indicated that they believed the program played an important and substantive role in their child’s success.

### Other Limitations

In addition to the lack of the control group, there are some other limitations with this “real-world” analysis that should be considered when interpreting these results. Parents articulated two difficulties in answering certain questions during the phone interview. Parents frequently stated that they had difficulty differentiating whether or not a certain outcome (eg, reduced number of accidents, improved school attendance) was due to the medications/laxatives their child was taking or due to U-CAN-POOP-TOO. More often than not in these cases, parents tended to assign most or all of the credit to the medication, making the findings reported here more conservative. Parents also noted that the questions did not take into account a change (decrease) in the volume of the accidents. Some parents stated that there was improvement but that this was not reflected in their answers as their child was still having accidents (just smaller accidents).

Another limitation of this paper relates to who was given the Web-based information prescription and the patients who were ultimately included in the analyses. Patients were not systematically identified or consecutively selected; instead, the physician used his own judgment as to whether a patient would be appropriate for receiving the Web-based information prescription. This judgment was based on the presentation of the family related to issues such as disorganization, apparent motivation, and readiness to change. This certainly limited the number of patients included but fits more appropriately with the notion of a “real world” prescription in that clinicians will likely provide Web-based information prescriptions to those whom they believe would benefit as opposed to providing it to everyone. It is not known what proportion of the overall clinic might have been deemed to benefit.

The final group used in the analyses was relatively small and all were Caucasian. These issues make it inappropriate to generalize these findings to a larger or more diverse population. Also, two of the 22 patients were not actually having accidents in the 2 weeks immediately prior to using the Internet intervention. They did, however, have a diagnosis of encopresis and reported accidents prior to this 2-week period. There is also an issue regarding the difference in time when patients used the program. Some patients accessed U-CAN-POO-TOO as long as 2 years before the interview, while others accessed it as recently as 2 months prior. However, in all the subanalyses conducted to examine this issue, no differences were found in any of the primary variables among groups of patients separated by varying enrollment dates.

Finally, not everyone who was given access to U-CAN-POOP-TOO used the program. Those who stopped using it for reasons other than resolution of their child’s encopresis identified lack of time and forgetfulness as two of the main barriers. This is consistent with our previous findings showing these as two of the most common barriers to families following through with Web-based information prescriptions [28]. However, it is important to reiterate that even given the number of patients who stated that they stopped using the system prior to the resolution of their problem, most of those had at least a 50% reduction in accident frequency. In addition, they indicated that the program had a substantive impact on symptom reduction. It is also worth noting that these patients might have appeared as “dropouts” in a clinical trial, but their perception is that the program made a difference in their care.

### Conclusions

This paper has important implications for the treatment of pediatric health problems using Internet interventions. These results indicate that parents believe Internet interventions can be helpful to their children outside of clinical trials. Although an increase in the development, testing, and use of Web-based applications is already occurring [32-34], this study lends additional support for the importance of this work. Given the potential for cost savings and the capability of large-scale dissemination of Internet programs, their appeal is obvious. While testing of the feasibility and efficacy of these types of interventions is increasing [35], this is, to our knowledge, the first “real world” study documenting patients’ perceived impact and utility of an Internet intervention in a pediatric population. Additional feasibility, efficacy, and real-world effectiveness studies are necessary to increase acceptance of Internet interventions and clearly establish their usefulness in the treatment of a variety of pediatric disorders.

## Abbreviations

BM: bowel movement
